# E-Cigarette Use Is Associated with Increased Psychological Distress among Youth: A Pooled Cross-Sectional Analysis of State-Level Data from 2019 and 2021

**DOI:** 10.3390/ijerph191811726

**Published:** 2022-09-17

**Authors:** Christopher Cambron

**Affiliations:** College of Social Work, University of Utah, Salt Lake City, UT 84112, USA; chris.cambron@utah.edu

**Keywords:** e-cigarettes, youth substance use, youth mental health, psychological distress

## Abstract

A crisis of worsening youth mental health in recent years across the United States has created alarm among health professionals. As a result, health professionals have sought to improve methods of identifying youth in need of treatment services. Cigarette, cannabis, and alcohol use each consistently serve as behavioral markers of risk for youth mental health problems. Despite the recent growth of electronic cigarette (e-cigarette) use among youth, few studies have examined whether e-cigarettes follow the same associational pattern with mental health problems in the context of other substance use. Additionally, the COVID-19 pandemic may have altered the associations between youth substance use and mental health problems due to both reduced overall use and increased mental health problems after the onset of the pandemic. The current study examined associations between youth substance use and psychological distress before and after the onset of the COVID-19 pandemic using two state-representative samples of youth in grades 8, 10, and 12 from 2019 (N = 58,689) and 2021 (N = 46,823) from Utah. Pooled cross-sectional linear and negative binomial regression models clustered by grade, stratified by school district, and weighted to represent population characteristics estimated associations between recent e-cigarette, combustible cigarette, cannabis, and heavy alcohol use and two measures of psychological distress—depressive symptoms and mental health treatment needs. After controlling for sociodemographic factors and recent uses of other substances, results indicated that psychological distress increased from 2019 to 2021 and that recent e-cigarette, combustible cigarette, cannabis, and heavy alcohol use were each significantly associated with increased levels on both measures of psychological distress. Compared to other substances, e-cigarette use showed the strongest standardized associations. The association of e-cigarette use with depressive symptoms strengthened significantly from 2019 to 2021. Given the youth mental health crisis paired with the widespread adoption of e-cigarettes, health professionals should consider recent e-cigarette use an increasingly important behavioral marker for risks of mental health problems among youth. Results suggest that future research studies examining the temporal ordering of substance use and mental health among youth should include e-cigarettes.

## 1. Introduction

Epidemiological surveillance in the United States indicates that as many as 1 in 5 youth suffer from serious mental health problems [1,2]. Numerous reports suggest that the incidence and severity of mental health problems have increased in recent years, resulting in a youth mental health crisis [2,3,4]. Additionally, millions of youth in the United States are unable to access formal mental health treatment services [2,4]. Failure to address serious mental health problems among youth can have lifelong consequences for health and wellbeing [5]. The youth mental health crisis in the United States was well established prior to the onset of the COVID-19 pandemic in March 2020. Reports indicate that COVID-19 negatively impacted youth mental health through multiple pathways including increased stress, reduced access to mental health treatment, social isolation, death of family members, and increased socioeconomic hardship, among others [6]. By December 2021, the Surgeon General of the United States issued a formal advisory highlighting the negative impact of COVID-19 on youth mental health [2]. This advisory called for the prioritization of both identification and treatment of youth mental health problems to stem the negative impacts of COVID-19 on an existing youth mental health crisis [2].

The bulk of evidence also suggests that youth substance use decreased after the onset of COVID-19 due to reduced social activities and limited social opportunities for use [7]. These trends may benefit youth, as substance use influences both short and long-term health and well-being [5]. In the short term, youth substance use is associated with risk for immediate harms such as unintentional injuries, traffic accidents, and self-harm. In the longer term, youth substance use is associated with increased incidence of mental health problems, psychiatric disorders, substance use disorders, and academic failure [8]. A range of studies have identified concurrent associations between youth substance use and mental health problems [5,8,9,10]. A systematic review of 48 longitudinal research studies noted clear associations between substance use and increased risk for mental health problems [10]. A more recent systematic review of studies on adolescent polysubstance use noted that 20 separate studies reported on the co-occurrence of youth substance use and mental health problems [9]. These studies could be categorized as positing three broad hypotheses to explain the consistent association between youth substance use and mental health problems—a common liability from the underlying risk driving both substance use and mental health problems, self-medication by youth experiencing psychological distress, and exacerbation of existing mental health problems by youth using substances [9]. This systematic review primarily focused on tobacco, cannabis, and alcohol and did not include studies on youth e-cigarette use.

Despite the introduction of e-cigarettes as a potential tool for cigarette smoking cessation, e-cigarette use among youth has grown substantially over the past decade in the United States. By 2016, the rapid increase in e-cigarette use prompted the Surgeon General to release a report describing the potential harms to youth [11]. In 2018, the Surgeon General declared e-cigarette use an epidemic among youth and recommended that parents and teachers take actions to reduce youth e-cigarette use [12]. By 2019, more than 40% of high school students reported lifetime e-cigarette use and 30% reported past year e-cigarette use [13]. While e-cigarettes are generally considered less harmful for youth than combustible cigarettes, cannabis, or alcohol, the liquid found in e-cigarettes often contains different combinations of nicotine, tobacco, heavy metals, or THC (tetrahydrocannabinol—the primary psychoactive compound in cannabis) [14]. All of these components of e-cigarette liquid present potential harms to youth to varying degrees, but longitudinal studies on physical health outcomes of e-cigarette use are limited by the recency of the epidemic among youth. In addition to direct harms on youth health, evidence suggests that youth e-cigarette use is reliably associated with subsequent initiation of both combustible cigarette and cannabis use [15,16,17].

Researchers have recently started to examine potential associations of e-cigarette use with youth mental problems [18]. Nationally representative data from 2013 to 2015 indicated that mental health problems were associated with increased risk for subsequent e-cigarette and combustible cigarette initiation among youth who had not previously used tobacco products [19]. Data from a community sample in California in 2013 and 2014 suggested a bi-directional association of e-cigarette use with youth mental health problems [20]. Results indicated that increased e-cigarette use was associated with increased future depressive symptoms, and increased depressive symptoms were associated with increased future e-cigarette use over time [20]. These results did not suggest that e-cigarettes may be causing mental health problems, but more likely the presence of a common underlying factor impacting both depressive symptoms and e-cigarette use. Cross-sectional analyses from this same community sample of California youth in 2013 characterized e-cigarette users as demonstrating a higher likelihood of multiple mental health problems compared to non-users [21]. E-cigarette users, however, showed fewer mental health problems than combustible cigarette users or dual e-cigarette/cigarette users [21]. A longitudinal study of a community sample from Philadelphia reported that depressive symptoms influenced future e-cigarette use but not vice versa from 2016 to 2019 [22]. One recent study did examine longitudinal associations from Fall 2018 to Fall 2020 between multiple substances and depressive symptoms among young adults age 18 to 34 recruited via social media [23]. Interestingly, this study reported that depressive symptoms before the onset of COVID-19 were associated with subsequent increases in cigarette and e-cigarette use, but cannabis and alcohol use did not follow these same patterns [23]. Results noted a high stability of e-cigarette use across the study period, which did not allow for an examination of how changes in e-cigarette use might influence changes in depressive symptoms. Importantly, this study did not test the unique contributions of e-cigarette, combustible cigarette, cannabis, or alcohol use before and after the onset of COVID-19 [23].

The current study is motivated by multiple features of previous research described above. First, the existing youth mental health crisis was likely exacerbated by the COVID-19 pandemic, resulting in greater levels of psychological distress. Across the onset of the pandemic, youth substance use dropped due to reduced social interactions. E-cigarettes are an increasingly popular form of substance use among youth and are likely associated with youth mental health problems. Despite these findings, few studies have included e-cigarettes when examining associations between the use of multiple substances and mental health problems among youth. Research has yet to examine if the use of different substances including e-cigarettes show independent associations with youth mental health problems before and after the onset of COVID-19. The current study addresses these questions by examining associations between youth e-cigarette, combustible cigarette, cannabis, and heavy alcohol use after accounting for sociodemographic factors in a state-representative sample of 8th, 10th, and 12th grade students from Utah in 2019 and 2021. The current study hypothesized that e-cigarette, combustible cigarette, cannabis, and heavy alcohol use would show independent associations with two indicators of youth mental health problems—depressive symptoms and mental health treatment needs. Dual reductions in substance use and increases in mental health problems among youth after the onset of COVID-19 likely altered associations between substance use and mental health problems.

## 2. Materials and Methods

### 2.1. Sample

This study used data from the 2019 and 2021 waves of the Prevention Needs Assessment (PNA) survey administered every two years to youth by the Utah Division of Substance Abuse and Mental Health (UDSAMH) [24]. The PNA provides similar items on youth substance use and health behaviors to the Center for Disease Control’s Youth Behavioral Risk Factor Surveillance System [25]. PNA data are clustered by grade, stratified by school district, and weighted to approximate the statewide demographics of youth. Ranking ratio estimation was used to ensure that the sample reflects the state population of students on a grade, gender, and race/ethnicity basis. Full details of the sampling strategy are available online from UDSAMH (https://dsamh.utah.gov/sharp-survey (accessed on 1 September 2022)). Parents or guardians of respondents provided active consent for participation. Validated and anonymized data for youth in grades 8, 10, and 12 from 2019 (N = 58,689) and 2021 (N = 46,823) were used for the current study. Table 1 provides sample characteristics.

### 2.2. Measures

#### 2.2.1. Psychological Distress

Depressive symptoms were measured by the mean of four questions: “Sometimes, I think that life is not worth it”, “At times, I think I am no good at all”, “All in all, I am inclined to think that I am a failure”, “In the past year, have you felt depressed or sad MOST days, even if you felt okay sometimes?” Each item had four Likert-style response options coded such that 0 = “definitely no” and 3 = “definitely yes”. Scores ranged from 0 to 3. Items originated from the Communities that Care survey, a valid and reliable instrument to measure youth risk and protective factors [26]. Mental health treatment needs were measured by the sum of six questions with the stem “During the past 30 days, how often did you feel…” followed by nervous, hopeless, restless or fidgety, so depressed that nothing could cheer you up, that everything was an effort, or worthless. Each item had five Likert-style response options coded such that 0 = “none of the time” and 4 = “all of the time”. Scores ranged from 0 to 24. Items originated in the K6, a valid and reliable instrument to measure moderate psychological distress and mental health treatment needs [27].

#### 2.2.2. Substance Use

Past 30-day e-cigarette and combustible cigarette use were each measured by a single item: “During the past 30 days, on how many days did you use (e-cigarettes, vape pens, or mods, or smoke cigarettes)?” with seven response options (0 days, 1 or 2, 3 to 5, 6 to 9, 10 to 19, 20 to 29, All 30 days). Cannabis use was measured by a single item “On how many occasions (if any) have you used marijuana (grass, pot, cannabis, weed) or hashish (hash, hash oil) during the past 30 days?” with seven response options (0, 1–2, 3–5, 6–9, 10–19, 20–39, 40+). Heavy alcohol use over the past 2 weeks was measured by a single item: “Think back over the last two weeks. If any, how many times have you had five or more alcoholic drinks in a row?” with six response options (none, once, twice, 3–5 times, 6–9 times, 10 or more times). Each substance use question was dichotomized such that 0 = “no use” and 1 = “any use”.

#### 2.2.3. Demographics

A single item measured each sociodemographic factor including age, gender, sexual orientation, race/ethnicity, and household education. Youth reported the highest level of schooling completed by an adult in their household. Responses were coded as high school or less, some college, college degree, and graduate degree. Gender provided response options of: Woman/Girl, Man/Boy, Transgender, and Other. Sexual orientation provided response options of: Heterosexual, Gay or Lesbian, Bisexual, or Not sure/Other. A binary measure of sexual or gender minority identity was calculated from both gender and sexual orientation items and included youth reporting as Transgender or Other gender and Gay or Lesbian, Bisexual, or Not sure/Other sexual orientation. Race/ethnicity provided response options of: American Indian or Alaskan Native, Asian, Black or African American, Hispanic/Latino, Native Hawaiian or other Pacific Islander, and White. Race/ethnicity categories were not mutually exclusive and were collapsed into two binary variables for non-White and Hispanic/Latino.

### 2.3. Analytic Plan

Data were analyzed with Mplus v8.8 [28]. Correlations were estimated for each year to examine the bivariate strength of association among variables. Pearson’s *r* was estimated for pairs of continuous variables, tetrachoric correlations for pairs of binary variables, polychoric correlations for pairs of ordered categorical and binary variables, and biserial correlations for pairs of continuous and binary or categorical variables. Depressive symptoms, mental health treatment needs, and age were treated as continuous in the correlation matrix, household education was treated as ordered categorical, and all other variables were treated as binary. Pooled, cross-sectional regression models were clustered by grade, stratified by school district, and weighted to approximate population characteristics. Each substance use variable was interacted with a binary indicator for year (0 = 2019, 1 = 2021) to assess potential changes in the strength of association across waves. Associations for depressive symptoms were estimated via linear regression, and all variables were entered simultaneously. Standardized effects allowing for direct comparison of the size of effect across independent variables measured on different scales were computed such that a one standard deviation unit change in each independent variable reflected a one standard deviation unit change in depressive symptoms. For binary independent variables, standardized estimates were computed such that a one unit change in the independent variable reflected a one standard deviation unit change in depressive symptoms. Associations with mental health treatment needs were estimated via negative binomial regression, given that the dependent variable could not result in counts of less than 0 (indicating no mental health treatment needs). Negative binomial regression is a generalization of Poisson regression approaches commonly used for count outcomes with a bounded floor of 0. Negative binominal regression relaxes the assumption of Poisson regression that the conditional mean and variance of the dependent variable are equivalent. This approach is more appropriate for overdispersed count outcomes and provides estimates on the log-rate scale that can be exponentiated to produce an incidence rate ratio (IRR) [29]. IRRs compare the risk rates across values of the independent variable, and IRRs of 1.0 indicate no difference in risk across levels of the independent variable. Alpha was set at 0.05, and diagnostics did not indicate any concerns regarding model assumptions [29]. Data were present for 96.2% of possible data points for 2019 and 95.4% of possible data points for 2021. Missing data were handled by full information maximum likelihood [28]. Multiple sensitivity tests were conducted to ensure the robustness of findings. Models estimating year specific effects separately produced substantively similar findings. Linear and Poisson regression models for mental health treatment needs produced substantively identical findings to those presented below indicating that results were robust to the choice of link function. Negative binomial models produced the most conservative results. Finally, models using unweighted data, year-specific weighting, and rescaled weights to reflect pooling of 2019 and 2021 data produced substantively identical results.

## 3. Results

Table 2 provides bivariate correlations among variables in the analytic sample and reports similar associations for 2019 and 2021 data. Table 3 provides results of pooled, cross-sectional regression models. A linear regression model for depressive symptoms found that Past 30-day e-cigarette, cigarette, and cannabis use and Past 2-week heavy alcohol use were each significantly associated with increased depressive symptoms (*p* < 0.001 for each). Standardized estimates ranged from 0.11 for e-cigarette use to 0.02 for combustible cigarette use. Youth in 2021 showed significantly increased depressive symptoms in 2021 compared to 2019 (Est. = 0.12, *p* < 0.001). The interaction of Past 30-day e-cigarette use by year yielded a significantly stronger association in 2021 compared to 2019 (Est. = 0.09, *p* = 0.047). Older, female, sexual/gender minority, and non-White youth each showed significantly higher depressive symptoms. Higher household education was significantly associated with lower depressive symptoms. A negative binomial regression model for mental health treatment needs showed a similar pattern of association as the model for depressive symptoms. Each substance use factor was significantly associated with increased mental health treatment needs (*p* < 0.001 for each), but no significant differences in the strength of association between each substance use factor and mental health treatment needs were noted from 2019 to 2021. IRRs ranged from 1.23 for e-cigarette use to 1.10 for cannabis use. Youth showed significantly increased mental health treatment needs in 2021 compared to 2019 (Est. = 0.10, *p* < 0.001). Older, female, sexual/gender minority, and non-White youth each showed significantly higher mental health treatment needs. Higher household education was significantly associated with lower mental health treatment needs.

## 4. Discussion

The current study examined associations between the use of multiple substances and youth mental health problems prior to and after the onset of the COVID-19 pandemic. Reports indicate that the pandemic led to both increases in mental health problems and reductions in substance use among youth [2,7]. Results of the current study mirrored those trends and suggest that e-cigarette use may serve as a behavioral marker of risk for mental health problems among youth above and beyond well-established associations between combustible cigarette, cannabis, and heavy alcohol use with youth mental health problems. Additionally, e-cigarette use showed the strongest standardized associations with two measures of psychological distress after accounting for the use of multiple other substances and sociodemographic differences. Results of the current study suggest that the stressors of the pandemic did not substantially alter associations between youth substance use and mental health problems among Utah youth. There is some evidence that the strength of association between e-cigarette use and depressive symptoms among youth may have increased after the onset of COVID-19. Given the widespread adoption of e-cigarettes among youth and the Surgeon General’s call to prioritize methods to identify youth at risk for mental health problems, current e-cigarette use should be recognized as a source of potentially important information about youth mental health. These results, however, should not be interpreted such that reductions in e-cigarette use among youth can help stem the youth mental health crisis. The clear association between e-cigarette use and two indicators of youth mental problems in the current study suggests that e-cigarettes should be included as one of a range of behavioral markers of risk for mental health problems.

Multiple other sociodemographic factors also showed significant associations with psychological distress after accounting for youth substance use. Older, female, sexual/gender minority, non-White, and lower-SES youth demonstrated significantly higher levels of both depressive symptoms and mental health treatment needs. Results of this study also suggest that special attention should be paid to the mental health of sexual and gender minority youth. A range of studies have consistently shown that sexual and gender minority youth are at increased risk for both mental health problems and substance use [30,31]. The current study highlights that the association between sexual and gender minority status and mental health problems persists after accounting for youth polysubstance use.

As mirrored by a systematic review of research on youth substance use after the onset of COVID-19, a lower percentage of youth in this study reported recent e-cigarette, combustible cigarette, cannabis, and heavy alcohol use in 2021 compared to 2019 [7]. Most youth engage in substance use in social settings with other school-age peers. Given school closures and reduced social activities during the height of the pandemic, some portion of students in the study likely had fewer opportunities to engage in substance use [32]. Additionally, specific to e-cigarettes and combustible cigarettes, the implementation of the Tobacco 21 law in July 2020 may have reduced the available supply of e-cigarettes and combustible cigarettes [33]. While youth in this study were not legally old enough to purchase tobacco products under Utah state law prior to Tobacco 21, the new law made it illegal for retailers to sell any tobacco, e-cigarette, or nicotine product to individuals under the age of 21. Prior to 2020, individuals aged 19 or older could purchase tobacco products in Utah, likely leading to an increased supply for high school age youth. Given the potentially reduced access to e-cigarette devices, youth in this study using THC-infused e-liquids may have also had fewer opportunities for use after the passage of Tobacco 21. Evaluations of the impact of Tobacco 21 on youth e-cigarette, combustible cigarette, and cannabis use can provide more insight into these questions. Despite these changes in the law, research has consistently shown that some youth are able to obtain e-cigarettes from older friends or family members as well as via internet-based sources [34].

Restrictions on social activities resulting from the pandemic may help explain the increased strength of association between youth e-cigarette use and mental health problems. With many school-age youth being forced to spend more time at home after the onset of the pandemic, parental norms may play an important role in substance use behaviors. E-cigarettes likely may be viewed by parents as less harmful compared to combustible cigarettes, cannabis, or alcohol [35]. As a result, youth seeking to self-medicate in response to psychological distress might encounter lower levels of discouragement from their parents or guardians [9]. In this sense, youth experiencing psychological distress that were self-medicating with cannabis or alcohol might be forced to substitute e-cigarettes while spending more time at home. The design of the current study cannot answer this question, but future studies should seek to understand substance substitution behavior among youth in different social contexts.

Some limitations to this study should be noted. First, only youth attending formal school were sampled by the PNA. As such, the current study could not account for youth who have dropped out of school. These youth may be at higher risk for both substance use and mental health problems [36,37] or may display different patterns of association between substance use and mental health problems. Second, Utah youth typically report lower substance use rates compared to other states, in part due to more restrictive social norms against substance use [38]. As such, results may not generalize to youth in other states. Third, the design of the current study cannot establish the presence of bi-directional associations between youth substance use and mental health problems over time or identify causal pathways and should not be interpreted as such [10]. Finally, while these results suggest youth experienced increased psychological distress from 2019 to 2021, these changes may be linked to multiple sources or stressors beyond the pandemic.

While improving the identification of youth in need of mental health treatment does not necessarily require a directionality of association, [2] future studies should examine potential bi-directional associations between youth use of multiple substances and mental health problems over time to potentially identify causal pathways. Appropriately designed longitudinal studies can provide support for directional or common liability hypotheses to explain associations between youth substance use and mental health problems. Findings from such studies can help health professionals target the earliest onset risk factors [9]. The bulk of current evidence on associations between the use of different substances and mental health problems, however, have not employed research designs capable of determining causality [9,10]. Pooled analyses of 15 longitudinal studies indicated that combustible cigarette use is likely bi-directionally associated with increased depressive symptoms among youth; but the path from depressive symptoms to future smoking appears stronger and more consistent [39]. Additional evidence from community samples indicates that combustible cigarette use may be more representative of the self-medication hypothesis among youth experiencing particularly high levels of psychological distress [31]. Preliminary evidence from cross-sectional samples of youth suggests that e-cigarettes may also operate in concordance with the self-medication hypothesis [40]. However, given the recency of the e-cigarette epidemic, longitudinal studies have yet to offer more definitive information on the directionality of associations between e-cigarette use and mental health problems among youth [40]. To date, there is no evidence suggesting that e-cigarettes are causing or exacerbating youth mental health problems. The strongest evidence from longitudinal birth cohort studies suggests that early initiation of and regular cannabis use among youth may increase risk for the development of mental health problems later in adolescence [41]. Additional evidence from systematic reviews suggests that youth engaged in heavy alcohol use are at greater risk for a range of future physical and mental health problems [42]. The currently available evidence primarily hypothesizes that youth cannabis and heavy alcohol use have the potential to alter brain development in ways that exacerbate underlying risks for the future onset of mental health problems [41,42]. It remains unclear if youth e-cigarette or combustible cigarette use can have such deleterious effects on youth brain development. Rigorous and innovative longitudinal research designs are needed to disentangle these complicated and potentially reciprocal pathways, both within each specific substance and across multiple substances.

## 5. Conclusions

Associations between youth cigarette, cannabis, and alcohol use and mental health problems are well established [9,10,39,41,42]. Given the substantial rise in e-cigarette use among youth in recent years, few studies have examined if e-cigarettes show the same pattern of association with psychological distress as other substances. The current study is among the first to examine associations between e-cigarette use and psychological distress among youth in the context of combustible cigarette, cannabis, and heavy alcohol use. Additionally, in light of reductions in substance-using behavior and increases in mental health problems after the onset of the COVID-19 pandemic [2,7], the current study is among the first to examine potential changes in these associations across the start of the pandemic. Results of pooled, cross-sectional regression models using state-representative data of 8th, 10th, and 12th grade youth from Utah (N = 105,512) suggest that recent e-cigarette, combustible cigarette, cannabis, and heavy alcohol use are independently associated with depressive symptoms and mental health treatment needs after accounting for sociodemographic factors. E-cigarette use showed the strongest associations, and the strength of association with depressive symptoms increased significantly from 2019 to 2021. Results suggest that health professionals may consider e-cigarette use in tandem with other substance use when seeking to identify youth at greater risk for mental health problems.

## Figures and Tables

**Table 1:** Sample characteristics and population estimates.

		2019 (N = 58,689)			2021 (N = 46,823)		
Variables		N	Unweighted M (SD), %	Weighted M (SD), %	N	Unweighted M (SD), %	Weighted M (SD), %
Grade							
	8	25,581	43.6	34.6	21,176	45.2	35.1
	10	20,376	34.7	33.8	16,374	35.0	33.2
	12	12,732	21.7	31.6	9273	19.8	31.7
Age		57,985	15.2 (1.6)	15.5 (1.7)	46,823	15.1 (1.6)	15.5 (1.7)
Sex/gender							
	Female	30,618	52.3	51.2	23,956	51.4	51.5
	Male	27,958	46.4	48.2	22,039	47.3	47.3
	Transgender/Other	769	1.3	0.6	567	1.2	1.2
Sexual orientation							
	Heterosexual	50,784	88.1	89.2	37,784	82.9	82.9
	Gay or Lesbian	892	1.5	1.4	902	2.0	1.9
	Bisexual	3136	5.4	5.1	3677	8.1	8.4
	Not sure/Other	2846	4.9	4.3	3222	7.1	6.8
Race/ethnicity							
	American Indian/Alaskan Native	1922	3.3	1.5	1091	2.3	1.2
	Asian	2160	3.7	2.4	1472	3.2	2.4
	Black/African American	1598	2.7	1.7	1069	2.3	1.7
	Hispanic/Latino	9702	16.5	18.7	6854	14.7	18.5
	Native Hawiain/Pacific Islander	1543	2.6	2	932	2.0	1.9
	White	47,614	81.1	76.5	39,379	84.6	77.3
Household education							
	High school or less	9760	18.4	20	6845	16.4	18.8
	Some college	7904	14.9	14.9	5347	12.8	13.3
	College degree	23,684	44.7	43.6	19,779	47.3	44.8
	Graduate degree	11,690	22	21.5	9881	23.6	23.1
Substance use							
	Past 30-day e-cigarette use	6489	11.1	12.4	3161	7.1	7.8
	Past 30-day cigarette use	831	1.4	1.5	477	1.0	1.0
	Past 30-day cannabis use	4419	7.2	8.1	2252	5.0	5.8
	Past 2-week heavy alcohol use	2265	3.9	4.4	1124	2.5	3.0
Psychological distress							
	Depressive symptoms	56,954	1.0 (1.0)	1.0 (1.0)	44,686	1.1 (1.0)	1.1 (1.0)
	Mental health treatment needs	52,094	7.8 (6.3)	7.8 (6.2)	40,603	8.5 (6.6)	8.8 (6.6)

*Note.* M = mean; SD = standard deviation; weighted %’s approximate population characteristics; Race/ethnicity and sexual orientation categories are not mutually exclusive.

**Table 2:** Bivariate correlations among variables in the analytic sample.

Variable		1	2	3	4	5	6	7	8	9	10	11	12
Depressive symptoms	1	-	0.78	0.40	0.32	0.35	0.28	0.06	0.30	0.43	0.14	0.15	−0.15
Mental health treatment needs	2	0.77	-	0.34	0.31	0.30	0.26	0.06	0.29	0.41	0.11	0.12	−0.14
Past 30-day e-cigarette use	3	0.32	0.28	-	0.77	0.85	0.73	0.14	0.12	0.28	0.18	0.21	−0.30
Past 30-day cigarette use	4	0.30	0.28	0.75	-	0.72	0.69	0.11	−0.01	0.30	0.05	0.08	−0.20
Past 30-day cannabis use	5	0.29	0.25	0.85	0.66	-	0.72	0.23	0.07	0.29	0.19	0.23	−0.26
Past 2-week heavy alcohol use	6	0.26	0.24	0.73	0.65	0.72	-	0.17	0.07	0.19	0.26	0.29	−0.25
Age	7	0.05	0.06	0.19	0.15	0.21	0.16	-	−0.02	−0.08	−0.06	−0.05	0.00
Female	8	0.25	0.24	0.05	−0.02	0.01	0.00	−0.02	-	0.35	−0.01	0.03	−0.03
Sexual/gender minority	9	0.36	0.35	0.20	0.30	0.23	0.19	−0.05	0.23	-	0.11	0.12	−0.13
Non-White	10	0.13	0.09	0.17	0.09	0.24	0.25	−0.03	0.01	0.07	-	0.90	−0.43
Hispanic/Latino	11	0.14	0.11	0.21	0.06	0.28	0.27	−0.03	0.04	0.08	0.88	-	−0.46
Household education	12	−0.15	−0.13	−0.26	−0.21	−0.27	−0.23	−0.02	−0.04	−0.12	−0.43	−0.47	-

*Note*. 2019 data (N = 58,689) are below the diagonal, and 2021 data are (N = 46,823) above the diagonal.

**Table 3:** Results of pooled, cross-sectional regression models for substance use and sociodemographic factors associated with depressive symptoms and mental health treatment needs for 2019 and 2021.

	Depressive Symptoms					Mental Health Treatment Needs				
Variables	Est.	SE	95% CI	Std.	*p*	Est.	SE	95% CI	IRR	*p*
Past 30-day e-cigarette use	0.36	0.04	0.29, 0.44	0.11	<0.001	0.21	0.02	0.16, 0.25	1.23	<0.001
Past 30-day cigarette use	0.21	0.05	0.12, 0.30	0.02	<0.001	0.13	0.03	0.07, 0.20	1.14	<0.001
Past 30-day cannabis use	0.17	0.03	0.12, 0.22	0.05	<0.001	0.10	0.02	0.06, 0.14	1.10	<0.001
Past 2-week heavy alcohol use	0.15	0.04	0.08, 0.22	0.03	<0.001	0.12	0.02	0.08, 0.17	1.13	<0.001
Year (2021 vs. 2019)	0.12	0.01	0.10, 0.14	0.06	<0.001	0.10	0.01	0.08, 0.12	1.11	<0.001
E-cigarette use *x* year	0.09	0.05	0.00, 0.18	0.02	0.047	0.01	0.03	−0.04, 0.07	1.01	0.640
Cigarette use *x* year	−0.09	0.08	−0.25, 0.07	−0.01	0.278	0.06	0.05	−0.05, 0.16	1.06	0.288
Cannabis use *x* year	0.03	0.06	−0.08, 0.14	0.01	0.606	0.00	0.04	−0.07, 0.07	1.00	0.946
Heavy alcohol use *x* year	−0.04	0.05	−0.13, 0.06	−0.01	0.439	−0.04	0.03	−0.10, 0.02	0.96	0.191
Age	0.04	0.01	0.02, 0.06	0.04	<0.001	0.05	0.01	0.03, 0.07	1.05	<0.001
Female	0.35	0.01	0.32, 0.37	0.18	<0.001	0.27	0.01	0.25, 0.29	1.31	<0.001
Sexual/gender minority	0.65	0.02	0.61, 0.70	0.23	<0.001	0.41	0.01	0.38, 0.43	1.50	<0.001
Non-White	0.11	0.02	0.07, 0.15	0.05	<0.001	0.03	0.02	0.00, 0.07	1.03	0.026
Hispanic/Latino	0.01	0.01	−0.02, 0.03	0.00	0.706	0.01	0.02	−0.02, 0.04	1.01	0.458
Household education	−0.06	0.01	−0.07, −0.05	−0.07	<0.001	−0.06	0.00	−0.06, −0.05	0.95	<0.001
Intercept	0.76	0.02	0.73, 0.79	-	<0.001	1.88	0.01	1.86, 1.90	-	<0.001

*Note.* N = 105,512; Est. = unstandardized estimate; SE = standard error; CI = confidence interval; Std. = standardized estimate; *p* = *p*-value; IRR = incident rate ratio; depressive symptoms estimates reflect linear regression, and mental health treatment needs estimates reflect negative binomial regression; estimates are clustered by grade, stratified by school district, weighted to reflect population characteristics; age was standardized prior to inclusion in the model.

## Data Availability

Data are available upon request from the Utah Department of Health.

## References

[1] Bitsko R.H., Claussen A.H., Lichstein J., Black L.I., Jones S.E., Danielson M.L., Hoenig J.M., Davis Jack S.P., Brody D.J., Gyawali S. (2022). Mental health surveillance among children—United States, 2013–2019. MMWR Suppl..

[2] Office of the Surgeon General (2021). Protecting Youth Mental Health: The US Surgeon General’s Advisory.

[3] Kalb L.G., Stapp E.K., Ballard E.D., Holingue C., Keefer A., Riley A. (2019). Trends in psychiatric emergency department visits among youth and young adults in the US. Pediatrics.

[4] Duong M.T., Bruns E., Lee K.A., Cox S., Coifman J., Mayworm A.M., Lyon A.R. (2021). Rates of mental health service utilization by children and adolescents in schools and other common service settings: A systematic review and meta-analysis. Adm. Policy Ment. Health Ment. Health Serv. Res..

[5] Catalano R.F., Fagan A.A., Gavin L.E., Greenberg M.T., Irwin CEJr Ross D.A., Shek D.T. (2012). Worldwide application of prevention science in adolescent health. Lancet.

[6] Jones E.A., Mitra A.K., Bhuiyan A.R. (2021). Impact of COVID-19 on mental health in adolescents: A systematic review. Int. J. Environ. Res. Public Health.

[7] Layman H.M., Thorisdottir I.E., Halldorsdottir T., Sigfusdottir I.D., Allegrante J.P., Kristjansson A.L. (2022). Substance use among youth during the COVID-19 pandemic: A systematic review. Curr. Psychiatry Rep..

[8] Office of the Surgeon General (2018). Facing Addiction in America.

[9] Halladay J., Woock R., El-Khechen H., Munn C., MacKillop J., Amlung M., Ogrodnik M., Favotto L., Aryal K., Noori A. (2020). Patterns of substance use among adolescents: A systematic review. Drug Alcohol. Depend..

[10] Macleod J., Oakes R., Copello A., Crome I., Egger M., Hickman M., Oppenkowski T., Stokes-Lampard H., Davey Smith G. (2004). Psychological and social sequelae of cannabis and other illicit drug use by young people: A systematic review of longitudinal, general population studies. Lancet.

[11] U.S. Department of Health and Human Services, U.S. Department of Health and Human Services (2016). E-Cigarette Use Among Youth: A Report of the Surgeon General.

[12] Office on Smoking and Health and National Center for Chronic Disease Prevention and Health Promotion (2018). Surgeon General’s Advisory on E-Cigarette Use among Youth. https://www.cdc.gov/tobacco/basic_information/e-cigarettes/surgeon-general-advisory/index.html.

[13] Johnston L., Miech R.A., O’Malley P.M., Bachman J.G., Schulenberg J.E., Patrick M.E. (2020). Monitoring the Future National Survey Results on Drug Use, 1975–2019.

[14] Centers for Disease Control and Prevention (2021). Smoking & Tobacco Use: Electronic Cigarettes. https://www.cdc.gov/tobacco/basic_information/e-cigarettes/index.htm.

[15] Harlow A.F., Stokes A.C., Brooks D.R., Benjamin E.J., Barrington-Trimis J.L., Ross C.S. (2022). E-Cigarette Use and Combustible Cigarette Smoking Initiation Among Youth: Accounting for Time-Varying Exposure and Time-Dependent Confounding. Epidemiology.

[16] Chan G.C.K., Stjepanović D., Lim C., Sun T., Shanmuga Anandan A., Connor J.P., Gartner C., Hall W.D., Leung J. (2021). Gateway or common liability? A systematic review and meta-analysis of studies of adolescent e-cigarette use and future smoking initiation. Addiction.

[17] Sun R., Mendez D., Warner K.E. (2022). Use of Electronic Cigarettes Among Cannabis-Naive Adolescents and Its Association With Future Cannabis Use. JAMA Netw. Open.

[18] Becker T.D., Arnold M.K., Ro V., Martin L., Rice T.R. (2021). Systematic review of electronic cigarette use (vaping) and mental health comorbidity among adolescents and young adults. Nicotine Tob. Res..

[19] Riehm K.E., Young A.S., Feder K.A., Krawczyk N., Tormohlen K.N., Pacek L.R., Mojtabai R., Crum R.M. (2019). Mental health problems and initiation of e-cigarette and combustible cigarette use. Pediatrics.

[20] Lechner W.V., Janssen T., Kahler C.W., Audrain-McGovern J., Leventhal A.M. (2017). Bi-directional associations of electronic and combustible cigarette use onset patterns with depressive symptoms in adolescents. Prev. Med..

[21] Leventhal A.M., Strong D.R., Sussman S., Kirkpatrick M.G., Unger J.B., Barrington-Trimis J.L., Audrain-McGovern J. (2016). Psychiatric comorbidity in adolescent electronic and conventional cigarette use. J. Psychiatr. Res..

[22] Moustafa A.F., Testa S., Rodriguez D., Pianin S., Audrain-McGovern J. (2021). Adolescent depression symptoms and e-cigarette progression. Drug Alcohol Depend..

[23] Wang Y., Duan Z., Romm K.F., Ma Y., Douglas Evans W., Bennett B., Fuss C., Klinkhammer K.E., Wysota C.N., Berg C.J. (2022). Bidirectional associations between depressive symptoms and cigarette, e-cigarette, cannabis, and alcohol use: Cross-lagged panel analyses among young adults before and during COVID-19. Addict. Behav..

[24] Utah Deparment of Human Services (2021). SHARP Survey.

[25] Centers for Disease Control Prevention (2019). Youth Risk Behavior Surveillance System (YRBSS). https://www.cdc.gov/healthyyouth/data/yrbs/index.htm.

[26] Arthur M.W., Hawkins J.D., Pollard J.A., Catalano R.F., Baglioni A.J. (2002). Measuring risk and protective factors for use, delinquency, and other adolescent problem behaviors: The Communities That Care Youth Survey. Eval. Rev..

[27] Prochaska J.J., Sung H.Y., Max W., Shi Y., Ong M. (2012). Validity study of the K6 scale as a measure of moderate mental distress based on mental health treatment need and utilization. Int. J. Methods Psychiatr. Res..

[28] Muthén B., Muthén L. (2017). Mplus.

[29] Tabachnick B.G., Fidell L.S., Ullman J.B. (2007). Using Multivariate Statistics.

[30] Russell S.T., Fish J.N. (2016). Mental health in lesbian, gay, bisexual, and transgender (LGBT) youth. Annu. Rev. Clin. Psychol..

[31] Rosario M., Schrimshaw E.W., Hunter J. (2011). Cigarette smoking as a coping strategy: Negative implications for subsequent psychological distress among lesbian, gay, and bisexual youths. J. Pediatric Psychol..

[32] Dumas T.M., Ellis W., Litt D.M. (2020). What does adolescent substance use look like during the COVID-19 pandemic? Examining changes in frequency, social contexts, and pandemic-related predictors. J. Adolesc. Health.

[33] Dai H., Chaney L., Ellerbeck E., Friggeri R., White N., Catley D. (2021). Rural-urban differences in changes and effects of Tobacco 21 in youth e-cigarette use. Pediatrics.

[34] Perikleous E.P., Steiropoulos P., Paraskakis E., Constantinidis T.C., Nena E. (2018). E-cigarette use among adolescents: An overview of the literature and future perspectives. Front. Public Health.

[35] Han G., Son H. (2022). A systematic review of socio-ecological factors influencing current e-cigarette use among adolescents and young adults. Addict. Behav..

[36] Maynard B.R., Salas-Wright C.P., Vaughn M.G. (2015). High school dropouts in emerging adulthood: Substance use, mental health problems, and crime. Community Ment. Health J..

[37] Tice P., Lipari R.N., van Horn S.L. (2017). Substance use among 12th grade aged youths, by dropout status. The CBHSQ Report.

[38] Burrow-Sánchez J.J., Ratcliff B.R. (2021). Adolescent Risk and Protective Factors for the Use of Electronic Cigarettes. J. Prev. Health Promot..

[39] Chaiton M.O., Cohen J.E., O’Loughlin J., Rehm J. (2009). A systematic review of longitudinal studies on the association between depression and smoking in adolescents. BMC Public Health.

[40] Gaiha S.M., Halpern-Felsher B. (2020). Escalating safety concerns are not changing adolescent E-cigarette use patterns: The possible role of adolescent mental health. J. Adolesc. Health.

[41] Scheier L.M., Griffin K.W. (2021). Youth marijuana use: A review of causes and consequences. Curr. Opin. Psychol..

[42] Lees B., Meredith L.R., Kirkland A.E., Bryant B.E., Squeglia L.M. (2020). Effect of alcohol use on the adolescent brain and behavior. Pharmacol. Biochem. Behav..

